# The Effects of Pet Ownership on Psychological Well-Being Among Socially Isolated Older Adults

**DOI:** 10.1093/geroni/igaa057.1033

**Published:** 2020-12-16

**Authors:** Tomoko Ikeuchi, Takumi Abe, Yu Taniguchi, Satoshi Seino, Yui Tomine, Chiho Shimada, Akihiko Kitamura, Shoji Shinkai

**Affiliations:** 1 Tokyo Metropolitan Institute of Gerontology, Tokyo, Japan; 2 Tokyo Metropolitan Institute of Gerontology, Tokyo, United States; 3 National Institute for Environmental Studies, Tsukuba, Ibaraki, Japan; 4 Tokyo Metropolitan Institute of Gerontology, Itabashi-ku, Japan

## Abstract

Accumulating evidence suggests that pet ownership associates with positive psychological outcomes (i.e., less loneliness, lower depression, etc.) in older adults. Yet, the role of pet ownership in psychological well-being (PWB) of socially isolated older adults is not fully explored. In this study, we hypothesized that pet (i.e., dog or cat) ownership would have positive effects on PWB among socially isolated older adults. The study used cross-sectional data of 9875 community-dwelling older adults collected in 2016 in a metropolitan area of Japan. Overall, 2841 (28.8%) participants were socially isolated (i.e., having social interactions with others less than once a week). Stratified by dog and cat ownership, 3143 (31.8%) were current or previous dog owners, and 1724 (17.5%) were current or previous cat owners. PWB was dichotomized using a score of the WHO-5 Well-Being Index, and 2730 (27.6%) were identified as low levels of PWB. Logistic regression models, adjusted for demographic confounders, showed that social isolation was associated with lower PWB (OR: 2.39; 95% CI: 2.17, 2.64) and lower odds of having a dog (OR: 0.70; 0.63, 0.77). When social isolation and dog ownership were entered into a model simultaneously as independent variables, dog ownership was associated with greater PWB (OR: 0.90; 0.81, 0.99). There was a significant partial mediating effect of dog ownership found on the association between social isolation and PWB (Sobel test p=.034). No such associations were observed in cat ownership. Our results suggest that having a dog may be effective for increasing PWB for socially isolated older adults.

